# Removal of Cr(VI) from Water Using a New Reactive Material: Magnesium Oxide Supported Nanoscale Zero-Valent Iron

**DOI:** 10.3390/ma9080666

**Published:** 2016-08-06

**Authors:** Alessio Siciliano

**Affiliations:** Department of Environmental and Chemical Engineering, Unversity of Calabria, Rende (CS) 87036, Italy; alessio.siciliano@unical.it; Tel.: +39-0984-496537

**Keywords:** chromium removal, kinetic tests, water purification, zero valent iron

## Abstract

The chromium pollution of water is an important environmental and health issue. Cr(VI) removal by means of metallic iron is an attractive method. Specifically, nanoscopic zero valent iron (NZVI) shows great reactivity, however, its applicability needs to be further investigated. In the present paper, NZVI was supported on MgO grains to facilitate the treatments for remediation of chromium-contaminated waters. The performances and mechanisms of the developed composite, in the removal of hexavalent chromium, were investigated by means of batch and continuous tests. Kinetic studies, under different operating conditions, showed that reduction of Cr(VI) could be expressed by a pseudo second-order reaction kinetic. The reaction rate increased with the square of Fe(0) amount, while it was inversely proportional to the initial chromium concentration. The process performance was satisfactory also under uncontrolled pH, and a limited influence of temperature was observed. The reactive material was efficiently reusable for many cycles without any regeneration treatment. The performances in continuous tests were close to 97% for about 80 pore volume of reactive material.

## 1. Introduction

Chromium is widely detected in surface water and groundwater because of its widespread application in metallurgy, organic chemical synthesis, leather tanning and wood preserving industries [1]. In the natural environment, chromium exists mainly in two oxidation states, Cr(VI) and Cr(III). Cr(VI) species, such as chromate (CrO_4_^2−^, HCrO_4_^−^) and dichromate (Cr_2_O_7_^2−^), are toxic, carcinogenic and highly soluble [2]. Contrarily, Cr(III) is relatively nontoxic and has low solubility at a wide pH range [2,3]. It does not readily migrate in groundwater since it usually precipitates as hydroxides, oxides or oxyhydroxides [1]. In the treatment of drinking waters and wastewaters, conventional techniques, including precipitation, phytoextraction, reverse osmosis, electrodialysis, ion exchange, membrane filtration and adsorption, have been tested and developed [4]. For the remediation of chromium-contaminated groundwater, the strategy of in situ reduction and precipitation/immobilization has largely been followed. Permeable reactive barriers (PRB) are suitable for removing several contaminants from groundwater [5,6,7,8,9,10]. Optimization of reactive materials is the major challenge in developing effective PRB technology [5]. Zerovalent iron (ZVI) has long been recognized as an effective material for environmental applications, because it is an excellent electron donor and because it is readily available, inexpensive and nontoxic [11,12,13]. Indeed, ZVI has been widely applied for the removal both of metals (chromium, lead, mercury, etc.), halogenated organic compounds (tetrachloroethene, trichloroethene, carbon tetrachloride, organochlorine pesticides and nitro aromatic compounds) and nitrate [11,14]. Compared to microscopic ZVI, the use of nanoscale zero-valent iron (NZVI) or nanoscopic bimetallic systems (i.e., Fe/Cu) to remediate groundwater could offer many advantages, such as an enhanced reactivity, by increasing the surface to volume ratios and providing more reactive surface sites [13,14,15,16,17,18,19,20]. Moreover, by exploiting NZVI, it is possible the direct injection of particles into subsurface water, in order to remediate the contaminants plume in the source zone [15]. However, in the absence of a stabilizing agent, NZVI particles exhibit a strong tendency to agglomerate into larger ones, due to the high surface energy and the intrinsic magnetic interaction, which causes the reduction of reactivity in application condition [15]. Furthermore, the remaining NZVI in the treatment zone makes the technology uneconomical and even generates secondary iron pollution [15]. To overcome these disadvantages, in recent years, it has been proposed to fix the NZVI particles onto proper materials. Several supports have been tested, including organic and inorganic materials: bentonite [1,4,21], zeolite [22,23], alginate [5], resin [24,25], silica fume [26], attapulgite [2], montmorillonite [27], organo-montmorillonite [28], pillared clay [29], palygorskite [30], rectorite [15], sepiolite [31], pumice [32], kaolin [33], chitosan [34], titanium nanotubes [35], carbon nanotubes [36], cellulose acetate [37]. Despite that these supports have proved their effectiveness for the treatment of a wide range of contaminants under experimental conditions, the development of new supporting materials remains a major issue in the application of NZVI processes [1]. Moreover, the mechanisms and the reaction kinetics are complicated and not well understood. In fact, different factors affect the reaction rate, such as the amount of both NZVI and contaminants, the pH and the presence of other oxidants, such as oxygen and nitrate [11]. In the present paper, a new NZVI supported material has been synthesized and applied for the removal of Cr(VI) in aqueous solutions. Specifically, due to its high capacity to establish intimate interactions with iron ions [38,39], MgO was used as the based material. This compound has several positive aspects. In fact, it is a common and cheap material, moreover, it can be eventually produced with different grain sizes, depending on the application necessities. These properties make its utilization particularly advantageous in PRB technologies. A two-step reduction technique was applied to successfully synthesize MgO-supported nanoscale zero-valent (MgO-NZVI) particles at normal pressure and temperature. Firstly, the iron ions were adsorbed on MgO grains, afterwards the MgO-NZVI particles were synthesized by reduction of Fe(III) ions with NaBH_4_. Several batch column experiments were carried out to evaluate the influence of initial Cr(VI) concentration, iron amount and temperature on the process efficiency. Furthermore, the effectiveness of developed material in continuous operation mode has been investigated. The reaction products have been identified and the process mechanisms were analyzed.

## 2. Results and Discussion

### 2.1. Reactive Material 

**Figure 1:**
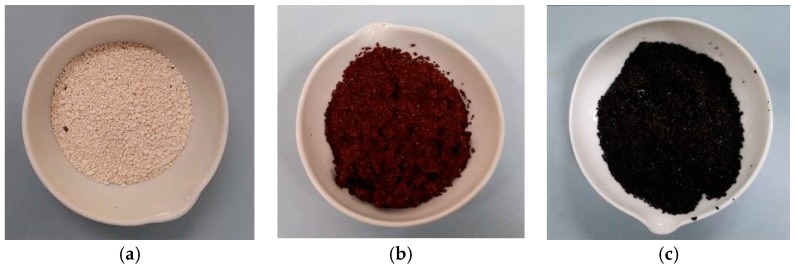
(**a**) MgO grains; (**b**) MgO grains after mixing with a solution of FeCl_3_·6H_2_O_2_; (**c**) MgO-NZVI after reaction with NaBH_4_.

**Figure 2:**
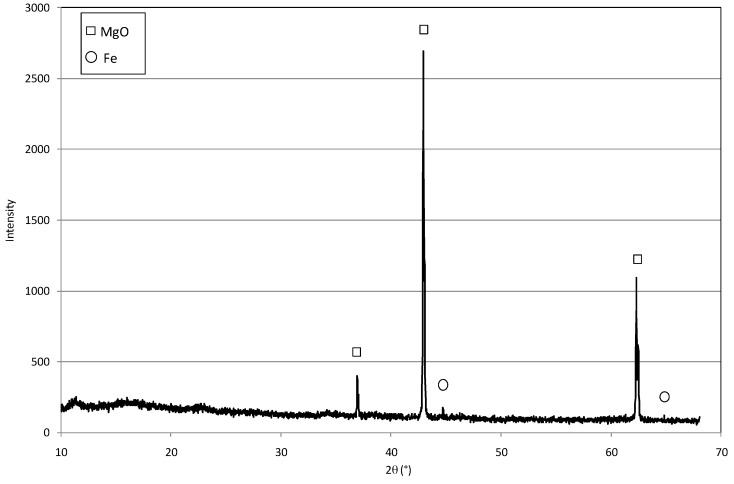
XRD (X-ray Diffraction) of MgO-NZVI particle.

**Figure 3:**
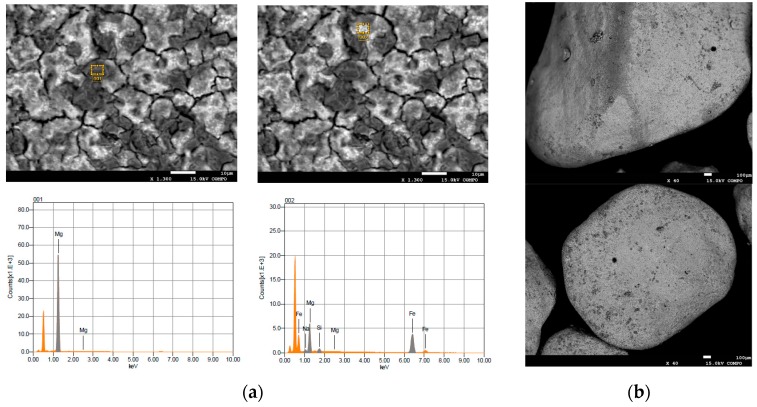
(**a**) EDX; and (**b**) SEM images of MgO-NZVI.

The MgO grains used in this study had a diameter ranging between 0.6 and 2 mm (Figure 1a). Figure 1 shows the prepared particles after each step of the defined procedure. The red-brownish color (Figure 1b) underlines the adsorption of Fe(III) ions on the surface of MgO grains, once the mixing of magnesium oxide with iron chloride was accomplished. The iron ions were then reduced to Fe(0) after the reaction with sodium borohydride, as the dark color of final material demonstrates (Figure 1c). The diffractogram confirms that Fe(0) nanoparticles were incorporated with the grains of magnesium oxide (Figure 2). The EDX (Energy Dispersive X-ray) analysis further underlines the presence of iron in the prepared composite (Figure 3). In particular, the Fe particles are mainly located in the light areas of image, while the dark zones are composed of magnesium oxide (Figure 3a). As can be noticed by the observation of SEM (Scanning Electron microscopy) images (Figure 3b), the zones characterized by the presence of iron particles are well distributed on the grains of MgO-NZVI. 

The mass fraction of NZVI in the MgO-NZVI particles, estimated according to the procedure reported in materials and methods section, was about of 7.5% (75 mg/g). This value is similar to that detected by Liu et al. [32]. The synthesized MgO-NZVI was determined to have a specific surface area of 24.41 m^2^/g, in line with the values reported in the literature for other types of reactive materials.

### 2.2. Batch Tests 

**Figure 4:**
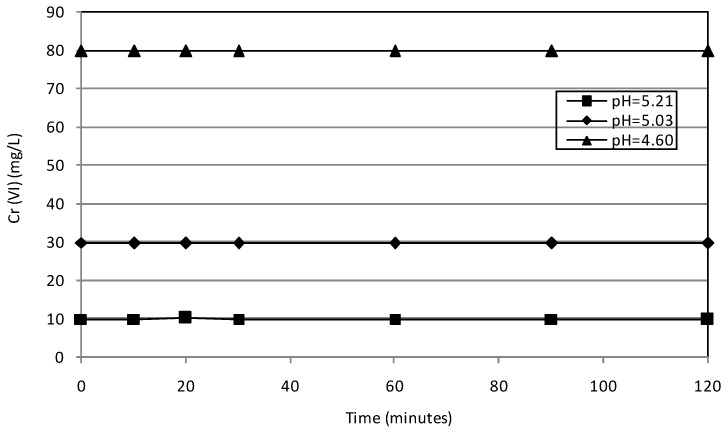
Cr(VI) trends during the tests conducted with unsupported MgO grains.

The first experiments were conducted to verify the ability of pure MgO particles in the removal of Cr(VI) from aqueous solutions. In this regard, 16 g of MgO were used to treat 300 mL of solutions characterized by Cr(VI) concentrations of 10 mg/L, 30 mg/L and 80 mg/L. The pH of these solutions was 5.21, 5.03 and 4.6, respectively. The detected results proved that the chromium abatement is not achievable with unsupported magnesium oxide particles. Indeed, despite the different values of initial concentration and pH tested, negligible reductions of Cr(VI) concentration were observed during a reaction time of about two hours (Figure 4). 

**Figure 5:**
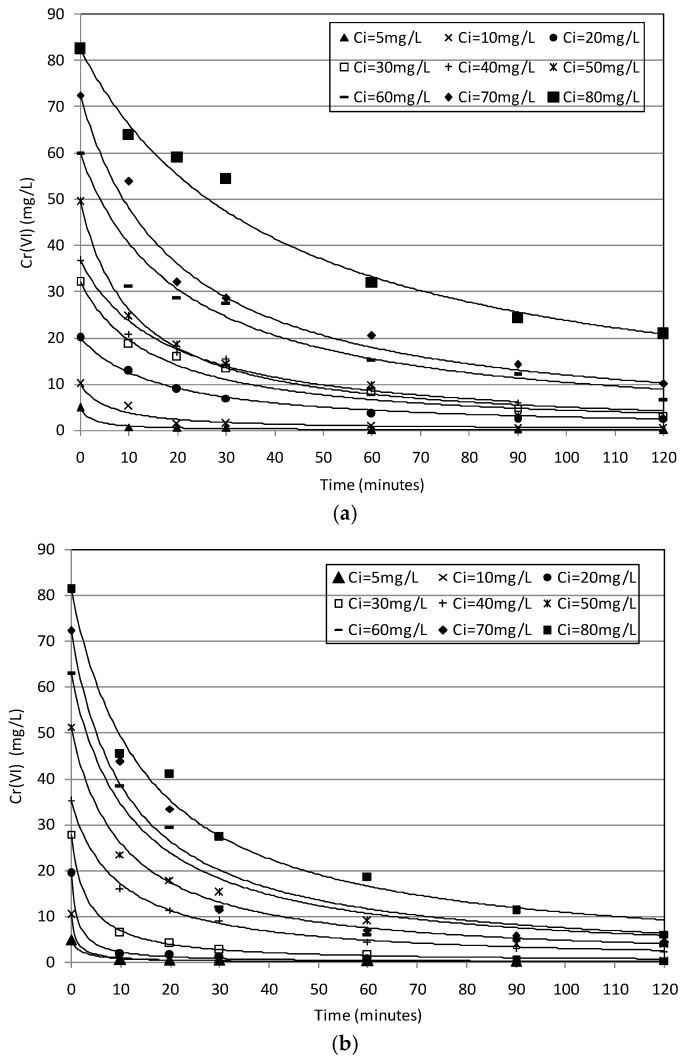

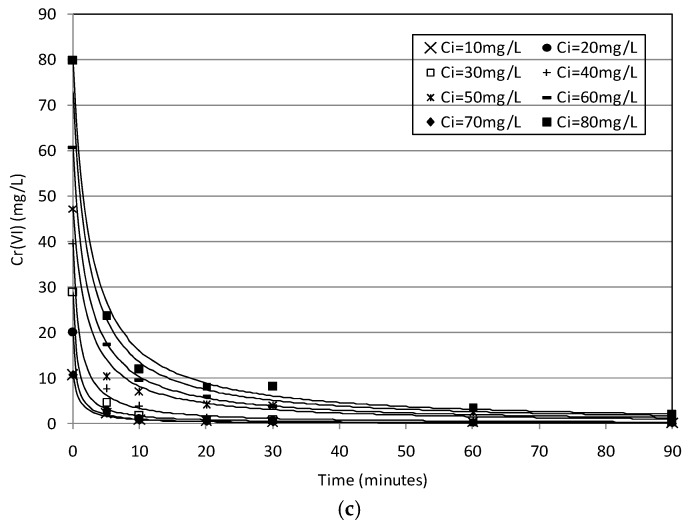
Cr(VI) removal trends detected during the tests conducted at *T* = 20 °C, using a MgO-NZVI amount of 8 g (**a**); 12 g (**b**); and 16 g (**c**). The *R*^2^ was equal to 0.99 for each modeled curve.

**Figure 6:**
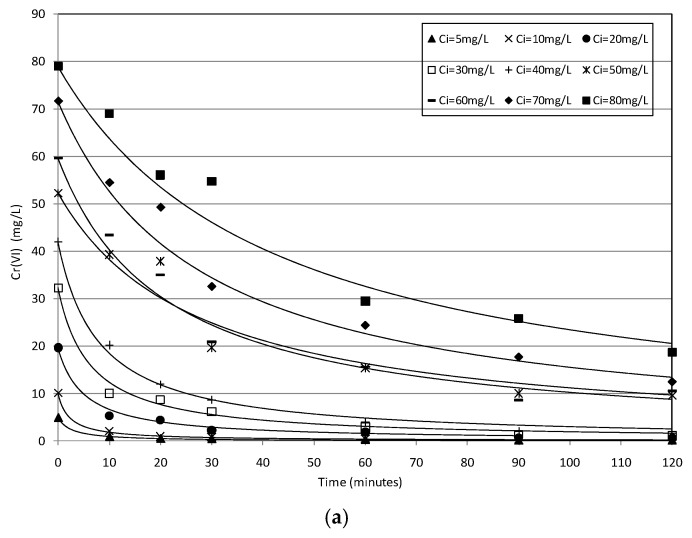

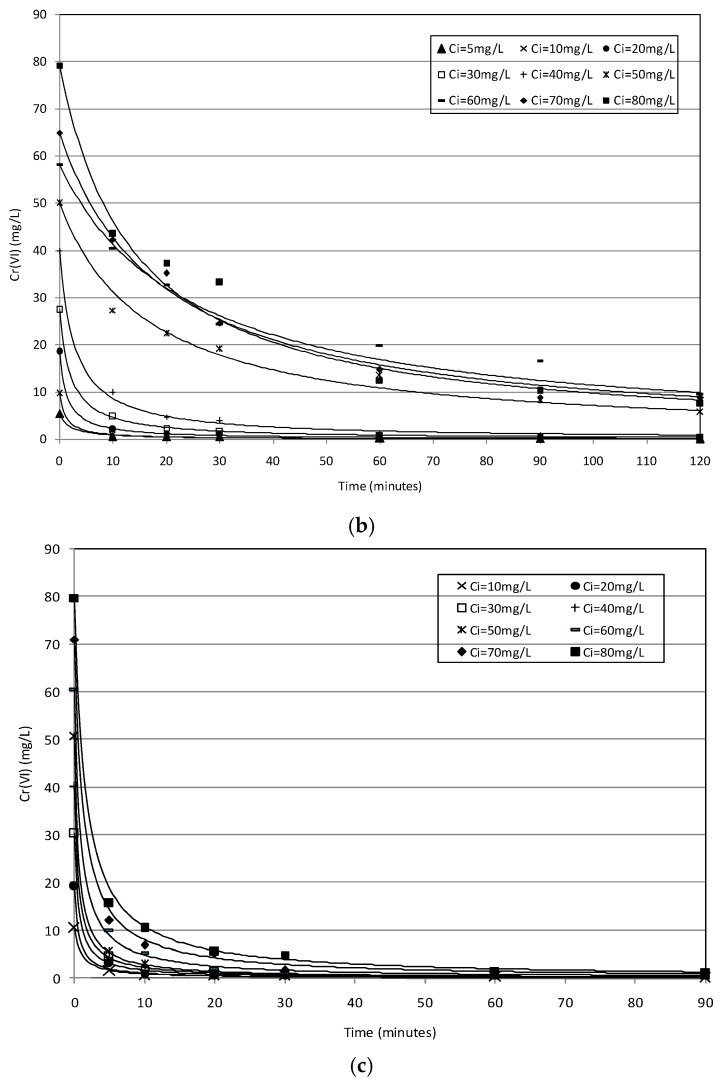
Cr(VI) removal trends detected during the tests conducted at *T* = 10 °C, using a MgO-NZVI amount of 8 g (**a**); 12 g (**b**); and 16 g (**c**). The *R*^2^ was equal to 0.99 for each modeled curve.

The next batch tests were conducted using supported MgO particles to treat standard solutions with chromium concentrations ranging between 5 and 80 mg/L and pH values between 4.6 and 5.3 (Table 2). In particular, for each concentration tested, MgO-NZVI amounts of 8 g, 12 g and 16 g were used. The corresponding actual amounts of Fe(0), by assuming the estimated percentage of iron equal to 7.5%, resulted of 0.6 g, 0.9 g and 1.2 g, respectively. The experimental results showed time decreasing curves typical of batch systems. In particular, the removal trends detected at 20 ± 1.5 °C, using 8 g of MgO-NZVI, have shown that a complete Cr(VI) abatement can be reached in about 30 min, by treating solutions with concentrations up to 10 mg/L (Figure 5a). Slower abatements were observed in response to the increase of the initial amount of chromium. Nevertheless, removals of about 90% were achieved in about two hours for Cr(VI) concentrations up to 40 mg/L (Figure 5a). Instead, an abatement of around 76% was obtained when the initial concentration was close to 80 mg/L (Figure 5a). Notable improvements of process performances were observed by increasing the amounts of MgO-NZVI. In fact, during the tests conducted with 12 g of reactive materials, faster reduction trends, at each operating condition were monitored (Figure 5b). By using amounts of MgO-NZVI equal to 16 g, for every initial Cr(VI) concentration, abatements greater than 88% were reached after only 10 min (Figure 5c). These results prove the efficiency in chromium reduction of developed reactive materials. Indeed, the performances detected in this work, taking into account the different operation conditions applied, are in line with those observed using others types of reactive materials and unsupported iron nanoparticles [1,20]. Moreover, the results of conducted experiments confirmed that the chromium removal is positively affected by the NZVI amount, while it decreases in response to initial Cr(VI) concentration of treating solution [24,34]. To investigate the process effectiveness at the typical temperature of groundwater, further batch tests were performed at a value of about 10 ± 1.5 °C. The detected results proved that the temperature had a relatively small effect on Cr(VI) removal by MgO-NZVI particles. Indeed, similar decreasing trends were observed at 20 °C and 10 °C, for each amount of reactive material and initial chromium concentration tested during the experiments (Figure 5 and Figure 6). These results are consistent with those detected by Kim et al. [22] using zeolite–nanoscale zero-valent iron composite as reactive material. Actually, chromium removal is a complicate process that includes phenomena that are differently affected by temperature. Specifically, the adsorption of Cr(VI) onto the metal surface and the transfer of electrons from the Fe(0) to the Cr(VI), resulting in Cr(III) as the major product, are generally considered the main process mechanisms. Temperature is an important factor that accelerates the chemical reactions but, instead, it would be generally expected that adsorption decreases with increasing temperature [22]. These opposite effects onto the mechanisms of chromium removal could explain the small influence of temperature on the overall process. Anyhow, contrary to the results of the present study, some works stated a greater positive influence of temperature on metals removal by means of nanoscale iron particles [4,34].

**Figure 7:**
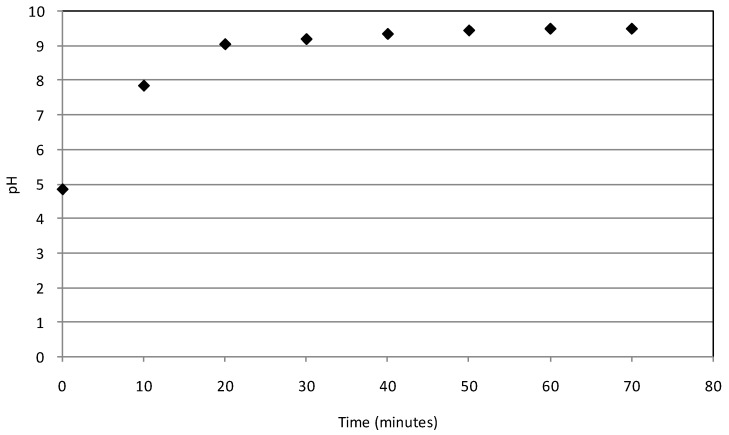
pH trend during the test conducted at 20 °C with 12 g of MgO-NZVI and initial Cr(VI) concentration of 50 mg/L.

The process pH is another factor that generally plays an important role during the removal of contaminants by iron. In fact, high removals, by means of Fe(0) application typically occur at acid conditions, because the iron corrosion is favoured and the precipitates’ formation, which would reduce the activity of iron particles, is avoided. Moreover, at acid pH, due to electrostatic attraction caused by the positive NZVI surface charge, the adsorption of chromium anion (CrO_4_^2−^, HCrO_4_^−^, Cr_2_O_7_^2−^) on reactive material is promoted. Different works stated the positive influence in maintaining acid conditions during the whole treatment period [20,31,34]. However, some researchers observed less influence of pH on metals’ removal by iron metal [1,22]. In the present work, no adjustment of pH during the process was performed and, for each operating condition tested, the experimental results showed rapid increasing trends from the initial pH values of standard solutions (Table 2) up to basic values. In Figure 7, a typical trend of pH during the Cr(VI) removal process is shown. This enhancement is attributable both to the natural iron corrosion in water and the Cr(VI) reduction process (Equations (1)–(4)). Anyhow, despite the rapid pH increase to alkaline values, remarkable chromium abatements were observed during the treatment.

**Figure 8:**
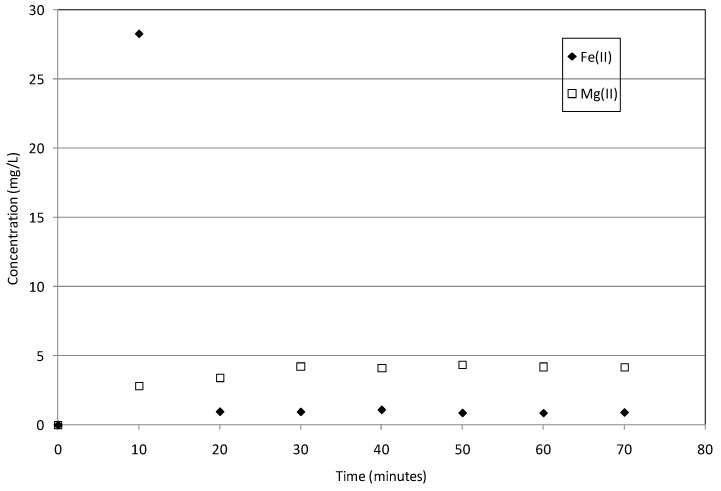
Fe(II) and Mg(II) concentrations detected during the test conducted at 20 °C with 12 g of MgO-NZVI and initial Cr(VI) concentration of 50 mg/L.

In fact, the chromium removal proceeds beyond the time required to reach basic pH and, thus, over the corrosion products’ precipitation (Figure 5 and Figure 6). These results, in agreement with Alowitz et al. [14] and Cheng et al. [40], suggest that also iron corrosion products may be responsible for appreciable contaminants’ reduction. Therefore, satisfactory performances can be reached regardless of the pH setting. Moreover, the basic pH reached during the process allows to reduce the dissolved iron concentration in the treated solutions. Indeed, the amount of iron ions increases during the initial minutes and then rapidly declines when the pH reaches values higher than 9. This is clear from the analysis of Figure 8, in which the concentrations of Fe(II) and Mg(II), which correspond to the pH trend showed in Figure 7, are reported. In general, in this study, the residual Fe(II) concentration was always lower than 1.5 mg/L for all the conducted experiments. This small iron amount is clearly a positive aspect, because it reduces the risk of secondary pollution phenomena in groundwater. Furthermore, only a low dissolution of magnesium ions was monitored during the chromium removal tests. In fact, despite the increasing Mg(II) concentration with the process evolution (Figure 8), maximum concentrations of about 5.0 mg/L were detected in the treated solutions. This restricted dissolution is attributable to the very low solubility of magnesium oxide. Moreover, the iron nanoparticles adsorbed on the surface limit the interactions of MgO grains with the treatment solutions. Anyhow, the residual values of Mg(II) are lower than the typical concentrations of natural waters. 

**Figure 9:**
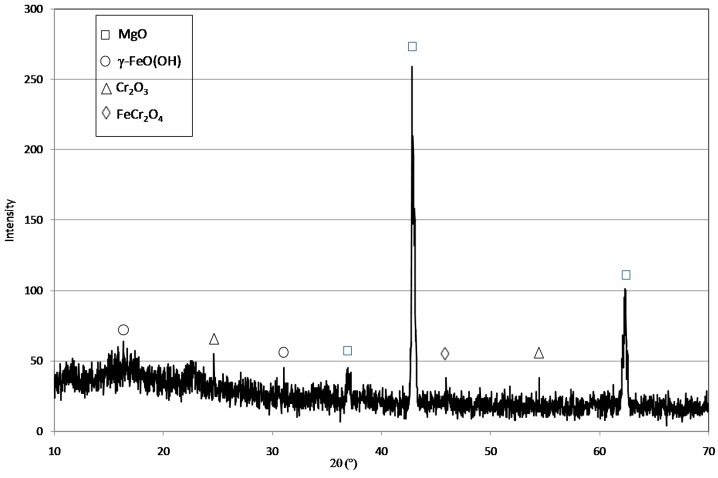
XRD of MgO-NZVI particles after the Cr(VI) reduction treatment.

**Figure 10:**
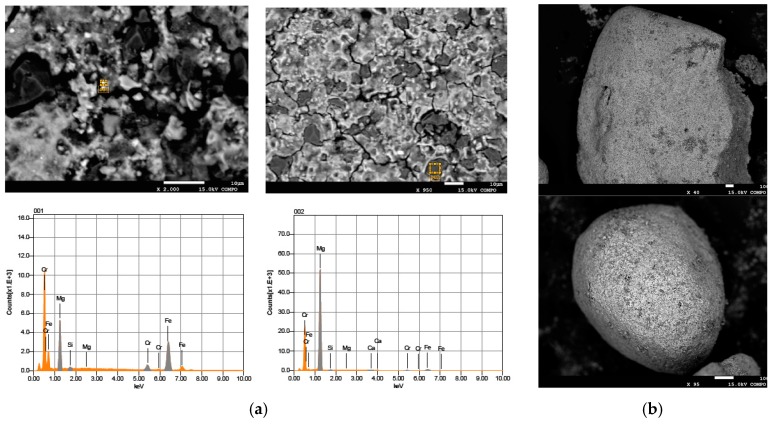
(**a**) EDX; and (**b**) SEM images of MgO-NZVI after the chromium removal treatment.

The XRD pattern of reactive material after reaction with Cr(VI) is illustrated in Figure 9. It can be noticed that the typical peaks of MgO became weaker, while peaks attributable to γ-FeO(OH), FeCr_2_O_4_ and Cr_2_O_3_ appeared. The EDX results confirmed that chromium was adsorbed on MgO-NZVI grains (Figure 10). In particular, it can be observed that the presence of Cr corresponds to that of Fe, while in the zones composed mainly of MgO (black zones), only a very low amount of chromium can be detected (Figure 10a). These observations demonstrated that the process products resulted from the adsorption and the redox reactions between Fe(0) and Cr(VI), where the supported NZVI acted as a reductant in aqueous solutions. 

Actually, the reduction of hexavalent chromium with ZVI can evolve according to several chemical reactions. When metallic iron is immersed in aqueous solutions, electrochemical corrosion will occur:

(1) $$2{\text{Fe}}^{0}+O_{2}+2H_{2}O\to 2{\text{Fe}}^{2+}+4{\text{OH}}^{-}$$

(2) $$4{\text{Fe}}^{2+}+4H^{+}+O_{2}\to 4{\text{Fe}}^{3+}+2H_{2}O$$

If Cr(VI) species are present, electrons are taken up by these species that become reduced:

(3) $$2{{\text{HCrO}}_{4}}^{-}+3{\text{Fe}}^{0}+14H^{+}\to 2{\text{Cr}}^{3+}+3{\text{Fe}}^{2+}+8H_{2}O$$

(4) $${{\text{HCrO}}_{4}}^{-}+3{\text{Fe}}^{2+}+7H^{+}\to {\text{Cr}}^{3+}+3{\text{Fe}}^{3+}+4H_{2}O$$

Under neutral or alkaline conditions, Cr(III) and iron species may be further removed from the aqueous system through precipitation as simple hydroxides:

(5) $${\text{Cr}}^{3+}+3{\text{OH}}^{-}\to \text{Cr}{\left(\text{OH}\right)}_{3}$$

(6) $${\text{Fe}}^{3+}+3{\text{OH}}^{-}\to \text{Fe}{\left(\text{OH}\right)}_{3}$$

or by co-precipitation as mixed oxides [41]:

(7) $${\text{Fe}}^{2+}+2{\text{Cr}}^{3+}+8{\text{OH}}^{-}\to {\text{FeCr}}_{2}O_{4}+4H_{2}O$$

Chromium and iron hydroxides may be further transformed in metal oxides with water release [41]:

(8) $$2\text{Cr}{\left(\text{OH}\right)}_{3}\to {\text{Cr}}_{2}O_{3}+3H_{2}O$$

(9) $$\text{Fe}{\left(\text{OH}\right)}_{3}\to \text{FeOOH}+H_{2}O$$

The XRD data of the present study are in agreement with the above mentioned mechanisms as well as with the results of other works [4,31].

### 2.3. Kinetic Analysis 

The above discussed results show how the chromium abatement through supported nanoscopic iron particles is affected by many factors. To investigate these impacts, a kinetic analysis was conducted. In this regard, the experimental removal trends were interpolated by using several kinetic laws. In general, a pseudo first order kinetic is properly used to simulate the removal processes of many type of contaminants [14,21,24,34,42]; anyhow, in this study, the best matching between the experimental results and the model predictions was obtained with the second order kinetic [43]:

(10) $$\frac{dC}{dt}=-K_{\text{obs}}\times C^{2}$$

here *C* is the Cr(VI) concentration and *K*_obs_ is the observed kinetic constant. By solving the above equation, the following expression for chromium concentration, as a function of time, can be easily obtained:

(11) $$C=\frac{C_{i}}{K_{\text{obs}}\times C_{i}\times t+1}$$

in which *C*_i_ represents the initial chromium concentration.

By means of the previous expression, it is possible to accurately simulate the results of the investigation carried out. Indeed, the remarkable agreement between the experimental results and theoretical predictions (Figure 5 and Figure 6) is a good validation of the proposed kinetic model. By interpolating the experimental trends, it was possible to estimate the values of the overall kinetic parameter (*K*_obs_) for each operating tested condition. It would be expected that, in this parameter, the effect of NZVI amount on reaction rate is included, while generally no dependence of kinetic constant from Cr concentration should occur.

**Figure 11:**
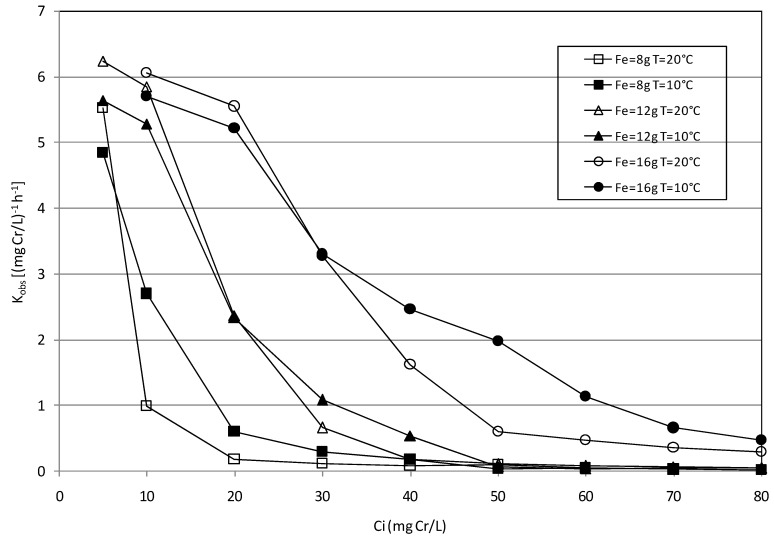
*K*_obs_ vs. initial chromium concentration for each Fe(0) amount and temperature tested during the batch tests.

Nevertheless, besides the expected positive influence of Fe(0) amount, the experimental results showed also the reduction of the reaction rates in response to the increase of initial Cr(VI) concentration. This is clear from the analysis of Figure 11, where the values of *K*_obs_ have been reported as a function of initial chromium concentration for each Fe(0) amount and temperature tested. These trends proved that the initial chromium amount affected the activity of the NZVI in agreement with the results reported by many other authors.

Specifically, Geng et al. [34] stated that, since Cr(VI) is a strong oxidant and a well-known passivator of Fe(0), as more Cr(VI) initial amount came close to the reactive materials, more Fe(0) would be oxidized and would lose its activity, leading to the decrease in the *K*_obs_. On the contrary, the increase of Cr(VI) reduction rates in response to the amount of nanoparticles is attributable to the corresponding increase in the total iron surface area and active sites [34]. The trends reported in Figure 11, as above mentioned, do not show a meaningful effect of temperature on chromium removal by means of MgO-NZVI particles. In general, it can be only noticed that the *K*_obs_ constants are slightly higher at 20 °C for the lower initial chromium concentrations, but these values decrease more rapidly than the kinetic constants observed at 10 °C. Anyhow, with the exception of some values, the trends at 10 °C and 20 °C are quite similar. However, it is not inconceivable that a greater impact on process performances could occur for temperatures higher than those tested in this study. 

By carefully analyzing the detected values of *K*_obs_, it was possible to identify a close dependence with the ratio between the square of iron amount and the initial chromium concentration (IC = Fe^2^/*C_i_*). In particular, a typical logistic trend which can be expressed by the following function of IC ratio can be identified:

(12) $$K_{\text{obs}}=\frac{K_{\text{max}}K_{0}e^{\beta \frac{{\text{Fe}}^{2}}{C_{i}}}}{K_{\text{max}}-K_{0}\left(1-e^{\beta \frac{{\text{Fe}}^{2}}{C_{i}}}\right)}$$

This expression includes the dependence of specific reaction rate from both the initial chromium concentration and Fe(0) amount. 

**Figure 12:**
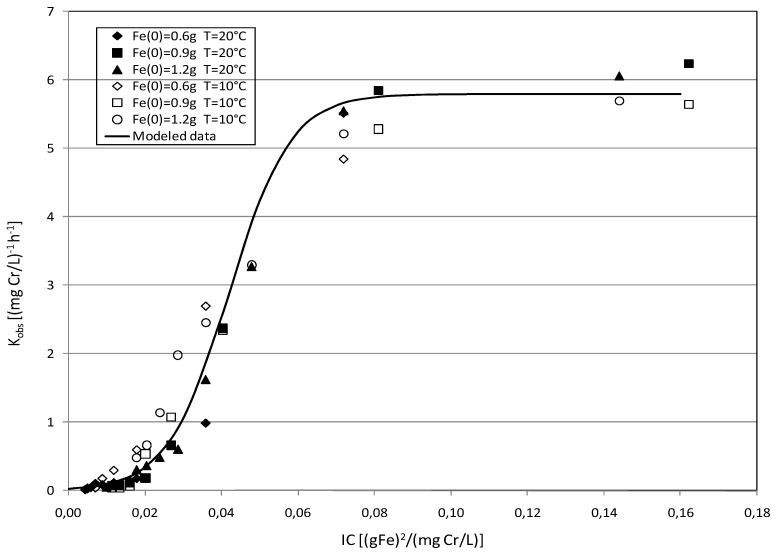
*K*_obs_ detected during the tests conducted at *T* = 20 °C and *T* = 10 °C with MgO-NZVI amounts of 8 g, 12 g and 16 g (the corresponding actual amounts of Fe(0) were 0.6 g, 0.9 g and 1.2 g) vs. Fe^2^/*C_i_* ratio.

The data reported in Figure 12 show that, for a given process temperature, the kinetic constants follow overlapping trends regardless of the iron amounts used in the tests. Moreover, for both the temperatures tested, the experimental trends can be quite well modeled with a single theoretical function, in which the *K*_0_, *K*_max_ and β values are equal to 0.03 (mgCr/L)^−1^·h^−1^, 5.82 (mgCr/L)^−1^·h^−1^ and 125 (gFe)^−2^/(mgCr/L)^−1^, respectively. This remarkable matching among the curves confirms that the IC ratio can be considered the main parameter affecting the process rate. 

Other works, instead, identified the Fe/*C_i_* ratio as the factor governing the Cr(VI) removal by means of iron nanoparticles [24]. The results of the present study clearly underline a much greater effect of the iron amount on the reaction rate. From the trends reported in Figure 12, it can be noticed that there is an exponential increase in the observed rate constant when the IC ratio is more than about 0.02 (gFe)^2^/(mgCr/L). Below this value, the reaction rates are significantly lower. According to Ponder et al. [24], it could be assumed that, at iron contents below this critical value, the surface sites are rapidly saturated and the mass transfer of contaminant to the occluded zero-valent iron becomes rate-limiting [24]. Nevertheless, also with the conditions to which the lowest values of K_obs_ correspond, a notable chromium removal, in restricted reaction time, can be obtained. The observed kinetic constant progressively increases up to reach an asymptotic value between 5 and 6 (mgCr/L)^−1^·h^−1^, for IC ratios higher than 0.07 (gFe)^2^/(mgCr/L). Therefore, for a given chromium concentration, a corresponding threshold iron value can be identified, beyond which the reaction rate is no longer affected by the amount of reactive material. 

### 2.4. Reuse of MgO-NZVI

**Figure 13:**
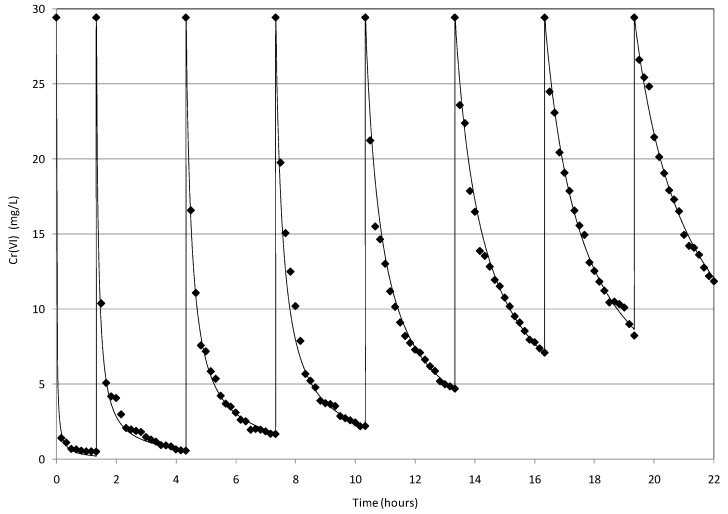
Cr(VI) removal trends detected during the repeated cycles conducted at *T* = 10 °C using a MgO-NZVI amount of 16 g for the treatment of solutions with Cr(VI) concentration of 30 mg/L.

The applicability of reactive material after many cycles is an important issue for practical applications. In the present study, the repeated use of MgO-NZVI for removing Cr(VI) was tested without the regeneration phase. The experimental results, obtained by repeatedly treating solutions with an initial concentration of 30 mg/L and pH of about 5.03, are shown in Figure 13. From the analysis of this graph, it can be primarily noticed that the second order kinetic model is able to accurately simulate the abatement curves detected in the subsequent treatment cycles. The experimental results clearly show a notable attenuation of reaction rates after the initial test. Indeed, the observed kinetic constant was reduced from the first value of 3.15 (mgCr/L)^−1^·h^−1^ to values lower than 0.47 (mgCr/L)^−1^·h^−1^ in the next treatment cycles (Table 1). This reduction of *K*_obs_ values is attributable to the progressive passivation of iron active surface. However, as discussed above, also in this condition it is possible to obtain a remarkable chromium removal in a moderate treatment period. In fact, in the first four subsequent tests, a Cr(VI) abatement higher than 90% was accomplished in about 3 h. This efficiency, during the repeated cycles, confirms that also the iron corrosion products are responsible for a certain chromium removal. After the 4th test, the process performance progressively decreased down to a value of about 60% in the 8th cycle. The results obtained in this study are in line with the statements of Li et al. [1], which observed an efficiency around to 95.5% in four subsequent treatment round, without regeneration of nanoscale zerovalent iron supported on pillared bentonite. Differently to the approach of the present study, other works tested the repeated applicability of reactive materials after a regeneration phase [4,31,32]. In particular, Liu et al. [32] observed a decrease of chromium removal from 76.5% to 45.5%, between the first and the fourth cycle, by means of the regeneration of nanoscale zero-valent iron supported on pumice. Fu et al. [31] stated the possibility to regenerate and reuse the resin supported NZVI obtaining removal efficiencies of Cr(VI), all above 80%. On the contrary, the results of Shi et al. [4] showed that, after a regeneration phase with EDTA, the ability of bentonite-supported nanoscale zero-valent iron to remove Cr(VI) was dramatically reduced after the first cycle. 

By comparing these statements with the findings of experiments conducted in the present work, it can be concluded that the reusability of MgO-NZVI, without a regeneration phase, makes it more practical in the field application for remediation of wastewaters and groundwater.

### 2.5. Continuous Tests

**Figure 14:**
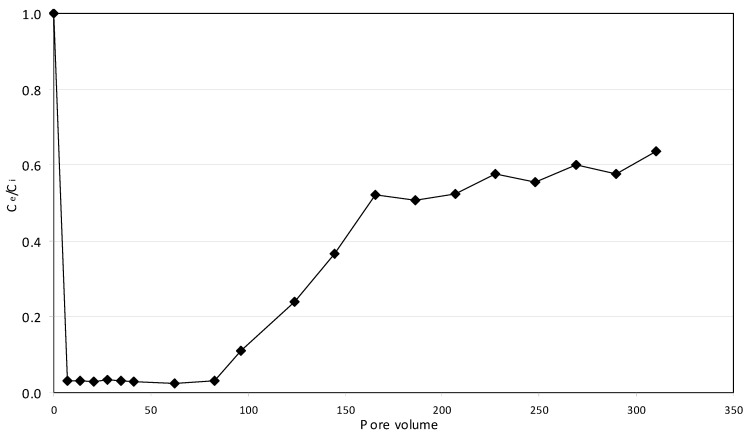
Normalized concentration in the effluent (*C_e_*/*C_i_*) vs. number of exchanged pore volume (PV) of reactive material.

The applicability of reactive materials in PRB technologies is determined by some important factors. In addition to the overall reaction rate, the ability to reduce a great amount of contaminant and the longevity of material are particularly important. Iron nanoparticles, with their surface area larger than that of microscopic iron, guarantee higher reaction rates, higher efficiency in terms of amount of contaminant reduced and long operation periods. However, the use of nanoparticles could adversely affect the permeability and thus could impede the flow of groundwater through an in situ permeable barrier [24]. The same problem could occur using NZVI supported on fine particles. The use of MgO-NZVI composite avoids the risk of clamping, because magnesium oxide particles can be produced at different grains size in order to achieve the appropriate porosity in situ applications. To verify the effectiveness of developed reactive material for PRB technologies, a continuous test was conducted using 32 g of MgO-NZVI for the treatment of a solution with a Cr(VI) concentration of 30 mg/L. The depth of reactive material was around 2 cm, the cross section was 5.72 cm^2^ and the porosity was about 0.57. The hydraulic flowrate was set to 2 mL/min and the resulting hydraulic velocity was about 8.86 m/d. A total volume of chromium solution of about 2 L was fed through the column, which corresponds to about 310 pore volume (PV) of reactive material. The variation of normalized Cr(VI) concentration in the effluent with treatment is shown in Figure 14. Despite that the flow velocity through the reactive material was much higher than the values commonly detected in groundwater [7], a satisfactory chromium removal was achieved. In particular, an abatement of about 97% was kept for nearly 80 pore volume (PV). Being the experiment conducted without deoxygenation of the solution fed through the column, the obtained results proved that the chromium abatement, by means of MgO-NZVI, efficiently evolves also in aerobic conditions. The observed performances are in line with the findings obtained by Zhang et al. [29], which used a combination of pillared bentonite with zero valent iron, as reactive material. Obviously, the ability to remove chromium could be easily increased by using smaller MgO grains for the preparation of reactive composite. In fact, by reducing the size of filling material, in order to decrease the porosity from the value of 0.57 (tested in this study) to about 0.30 (typical for the sands), the amount of active NZVI would approximately double. This, correspondingly, would allow greatly increasing the overall volume of contaminated water that could be treated in a permeable reactive barrier. 

## 3. Materials and Methods

### 3.1. Chemicals

Magnesium oxide (MgO), iron chloride hexahydrate (FeCl_3_·6H_2_O), sodium borohydride (NaBH_4_) and potassium dichromate (K_2_Cr_2_O_7_) were used during the experiments. All chemicals were of analytical grade and used without further purification.

### 3.2. Preparation of Reactive Material

The MgO-supported nanoscale zero-valent (MgO-NZVI) particles were synthesized by the following two steps procedure. During the first phase, 32 g of MgO were added to a ferric solution made by dissolving 33.7 g of FeCl_3_·7H_2_O_2_ into 180 mL of ethanol plus 70 mL of deionized water (0.5 M of Fe). The mixture was mechanically stirred at 450 rpm, over 24 h, at room pressure and temperature. The resulting particles were recovered by filtering and were washed with deionized water. In this phase, the Fe(III) was efficiently adsorbed on MgO grains. In the second step, the reduction of ferric ions to Fe(0) was accomplished by means of sodium borohydride [16] which promotes the following reaction:

(13) $$4{\text{Fe}}^{3+}+3{{\text{BH}}_{4}}^{-}+9H_{2}O\to 4{\text{Fe}}^{0}+3H_{2}{{\text{BO}}_{3}}^{-}+12H^{+}+6H_{2}↑$$

Specifically, the prepared particles were dispersed in a solution obtained by dissolving 9.45 g of NaBH_4_ powder in 180 mL of ethanol and 70 mL of deionized water (1 M of B). The mixture was mechanically stirred at 250 rpm, at room conditions for 2 h. Afterwards, the mixture was centrifuged for 30 min at 4000 rpm and the recovered solid was washed twice with deionized water. The prepared wet MgO-NZVI particles were used immediately for chromium removal tests.

### 3.3. Column Tests

**Figure 15:**
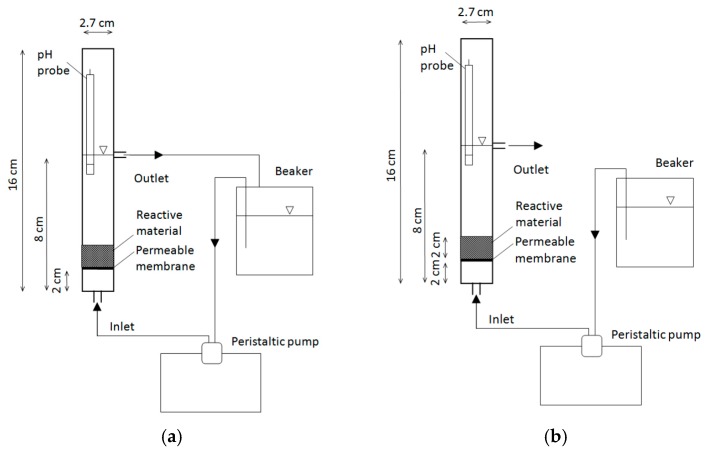
Scheme of the system used for (**a**) batch tests; and (**b**) continuous test.

The experiments were performed on a small laboratory equipment, consisting of a plexiglass cylinder characterized by a diameter of 2.7 cm and an overall height of 16 cm (Figure 15). Two 0.6 cm holes were made, respectively, at the bottom of the column and at a height of about 8 cm, for the inlet and for the outlet flow, respectively (Figure 15). The system was provided with a permeable plexiglass membrane, placed at 2 cm from the bottom, to bolster the reactive material (Figure 15). 

Several batch tests were conducted to investigate the influence of initial Cr(VI) concentration, reactive material amount and temperature on process performance. In this regard, a total of 52 tests were conducted at 10 ± 1.5 °C and 20 ± 1.5 °C, using wet MgO-NZVI amounts of 8 g, 12 g and 16 g (the thicknesses in the column were of about 0.5 cm, 0.75 cm and 1 cm) for the treatment of 300 mL of standard solutions with initial chromium concentrations comprised between 5 and 80 mg/L (Table 2). Moreover, some specific tests were conducted to verify the potential chromium abatement of pure unsupported MgO grains. In particular, 16 g of magnesium oxide were used to treat standard solutions characterized by Cr(VI) concentration equal to 10 mg/L, 30 mg/L and 80 mg/L. During the batch tests, the reactive material was placed on the permeable membrane and the Cr(VI) solution was recirculated in up-flow mode through the column by means of a peristaltic pump (Figure 15a). The flow-rate was set to 52 mL/min in order to ensure a very quick recirculation of chromium solution and avoid mass transfer limitations. By considering the amounts of the reactive material, the volume of the treating solution and the operating conditions applied, the maximum conversion per pass cannot exceed the 0.5% of total chromium mass. This assures that the reactor behaves like a time differential reactor to be assimilated to an actual batch system.

The temperature of standard chromium solution was initially set to the established value and then the experimental equipment was placed in a thermostatic refrigerator for the test execution. 

After this set of experiments, a series of successive batch tests was performed to analyze the behavior of reactive material in treating subsequent amounts of chromium. Then, 300 mL of standard solution, with an initial Cr(VI) concentration of 30 mg/L, was repeatedly treated during these tests. The amount of reactive material was set to 16 g. 

The process performance in continuous operation mode was investigated by means of a further experiment, conducted using 32 g of MgO-nZVI as reactive material. About 2 L of a standard solution, with initial concentration of 30 mg/L, was fed at a flow-rate of 2 mL/min in up-flow mode through the column (Figure 15b). 

All experiments were conducted without pH adjustment and no attempts were made to exclude oxygen from the treating solutions. During the tests, samples of 5 mL were periodically taken and, after a filtration at 0.45 μm, they were suddenly analyzed with respect to the amount of chromium and iron ions. 

### 3.4. Analytical Methods and Presentation of Results

Specific surface area (BET surface area) of MgO-NZVI particles was measured using the micrometric N_2_ adsorption method. The samples were degassed at 300 °C under vacuum prior to the measurement.

The iron amount incorporated on reactive material was estimated through the analytic determination of elements in the solution obtained by dissolving, at 60 °C, 0.5 g of MgO-NZVI into 50 mL of sulfuric acid (1:1). X-ray diffractometry (XRD) and scanning electron microscopy (SEM-EDX) were used to characterize the materials before and after the Cr(VI) removal treatment. During each test, temperature, pH and dissolved oxygen were measured by a multiparametric probe placed inside the column (Figure 15); the ionic forms Cr(VI) and Fe(II) were determined by colorimetric methods using an UV spectrophotometer, while Mg(II) concentration was estimated by means of atomic adsorption method [44]. Each measurement was carried out in quadruplicate and the mean value was reported. The relative standard deviation was always less than 5%. 

## 4. Conclusions

In this research, a new reactive material, composed of iron nanoparticles supported on MgO grains (MgO-NZVI), has been successfully developed. The specific surface area was about 24.41 m^2^/g and the Fe(0) content was around 75 mgFe/g. The results of the tests carried out proved the effectiveness of the synthesized reactive material in the removal of Cr(VI), under different operating conditions. By means of a kinetic analysis of the detected removal trends, a second order kinetic-type reaction was identified for each operating condition tested. The observed kinetic constant, for a given process temperature, resulted in a logistic-type function of the Fe^2^/C_i_ ratio. This demonstrated that the increase of Fe(0) dosage clearly produces a great enhancement of the chromium abatement, while the initial Cr(VI) amount of treating solution negatively affected the reduction process. On the contrary, a small influence of temperature was observed. In fact, similar chromium decreasing trends at 10 °C and 20 °C were detected. The chromium removal by means of MgO-NZVI composite efficiently evolved in aerobic conditions and without pH control as well. Furthermore, due to the basic conditions that were rapidly reached during the process, extremely low amounts of iron ions in the treated solutions were obtained. The characterization of reactive material before and after the reaction with chromium allowed identifying the γ-FeO(OH), FeCr_2_O_4_ and Cr_2_O_3_ as the main reaction products. The reaction mechanisms have been analyzed and proposed. The conducted experiments also proved the applicability of reactive material after many treatment cycles. In fact, Cr(VI) abatements, greater than 90%, were accomplished in about 3 h during four subsequent tests. Satisfactory process performances were achieved also in continuous treatments. Indeed, chromium abatements, near to 97%, were kept for about 80 pore volume of reactive material. In conclusion, MgO-NZVI composites could provide a valid opportunity for treatment of water contaminated by chromium. Anyhow, further experiments are necessary to verify the applicability of the process under field conditions.

## Figures and Tables

**Table 1:** Values of *K*_obs_ detected during the repeated cycles.

Cycle	1	2	3	4	5	6	7	8
*K*_obs_ (mgCr/L)^−1^·h^−1^	3.15	0.47	0.18	0.13	0.06	0.035	0.027	0.018

**Table 2:** Cr(VI) concentrations and pH values of the solutions treated during the batch tests.

Parameter	Values								
Cr(VI) mg/L	5	10	20	30	40	50	60	70	80
pH	5.29	5.21	5.12	5.03	4.94	4.85	4.77	4.69	4.60
